# Delayed Graft Function After Kidney Transplantation: The Role of Residual Diuresis and Waste Products, as Oxalic Acid and Its Precursors

**DOI:** 10.3389/ti.2024.13218

**Published:** 2024-07-19

**Authors:** Gideon Post Hospers, Wesley J. Visser, Jeroen G. H. P. Verhoeven, Mirjam Laging, Sara J. Baart, Ingrid R. A. M. Mertens zur Borg, Dennis A. Hesselink, Anneke M. E. de Mik-van Egmond, Michiel G. H. Betjes, Madelon van Agteren, David Severs, Jacqueline van de Wetering, Robert Zietse, Michel J. Vos, Ido P. Kema, Marcia M. L. Kho, Marlies E. J. Reinders, Joke I. Roodnat

**Affiliations:** ^1^ Department of Internal Medicine, Nephrology and Transplantation, Erasmus MC Transplant Institute, University Medical Center, Rotterdam, Netherlands; ^2^ Department of Internal Medicine, Division of Dietetics, Erasmus MC, Rotterdam, Netherlands; ^3^ Department of Epidemiology and Biostatistics, Erasmus MC, Rotterdam, Netherlands; ^4^ Department of Anesthesiology Erasmus MC, Rotterdam, Netherlands; ^5^ Department of Clinical Chemistry Metabolic Diseases, University Medical Center, Groningen, Netherlands

**Keywords:** delayed graft function, kidney transplantation, residual diuresis, accumulated waste products, oxalic acid, glyoxylic acid, deceased donor, living donor

## Abstract

Delayed graft function (DGF) after kidney transplantation heralds a worse prognosis. In patients with hyperoxaluria, the incidence of DGF is high. Oxalic acid is a waste product that accumulates when kidney function decreases. We hypothesize that residual diuresis and accumulated waste products influence the DGF incidence. Patients transplanted between 2018–2022 participated in the prospective cohort study. Pre-transplant concentrations of oxalic acid and its precursors were determined. Data on residual diuresis and other recipient, donor or transplant related variables were collected. 496 patients were included, 154 were not on dialysis. Oxalic acid, and glyoxylic acid, were above upper normal concentrations in 98.8%, and 100% of patients. Residual diuresis was ≤150 mL/min in 24% of patients. DGF occurred in 157 patients. Multivariable binary logistic regression analysis demonstrated a significant influence of dialysis type, recipient BMI, donor type, age, and serum creatinine on the DGF risk. Residual diuresis and glycolic acid concentration were inversely proportionally related to this risk, glyoxylic acid directly proportionally. Results in the dialysis population showed the same results, but glyoxylic acid lacked significance. In conclusion, low residual diuresis is associated with increased DGF incidence. Possibly accumulated waste products also play a role. Pre-emptive transplantation may decrease the incidence of DGF.

## Graphical Abstract

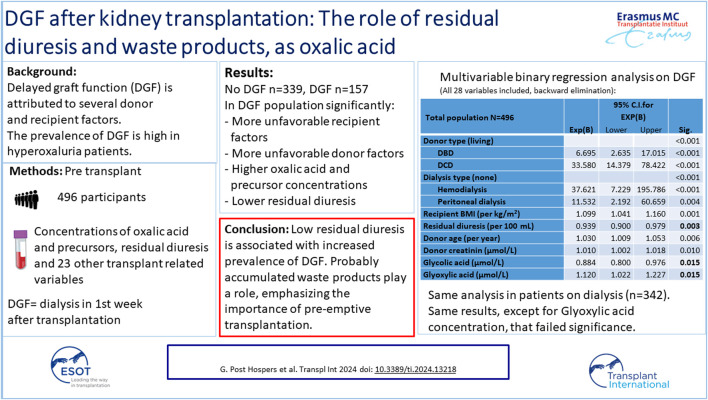

## Introduction

Delayed graft function (DGF) after kidney transplantation has been associated with donor, recipient and transplant related factors [1–3]. However, DGF most probably is multifactorially determined while the contribution of those factors varies in different studies. DGF occurs in about 30% of recipients of a deceased donor organ [2, 3] and 3.6% of recipients of a living donor kidney [1]. DGF is associated with decreased short and long term graft survival, partly due to an increased rejection risk [4, 5]. This means that DGF should be prevented when possible.

Acute tubular necrosis (ATN) in DGF differs from native kidney ATN in several ways, but the most striking is the prevalence of polarizable crystals consistent with calcium oxalate in DGF [6]. Calcium oxalate deposition in the transplanted kidney heralds a bad prognosis [7–9]. Experience with transplantation in patients with primary or secondary hyperoxaluria demonstrated that this population had higher rates of DGF, partially accompanied by biopsy-proven calcium oxalate deposition, compared to the non-hyperoxaluria population [10].

**FIGURE 1:**
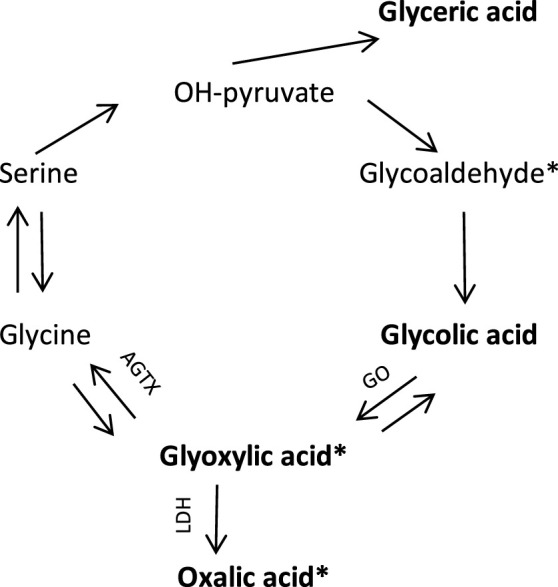
Endogenous oxalic acid synthesis pathway [11]. In bold are the substances whose concentrations were determined in this study. * known to be nephrotoxic, tubulotoxic [12, 13]. GO, glycolate oxidase; AGTX, Alanine glyoxylate aminotransferase; LDH, Lactic dehydrogenase.

Primary hyperoxaluria is a group of autosomal recessive genetic disorders of the glyoxylate metabolism (Figure 1) [14, 15]. Oxalic acid is the end product of many metabolic processes and cannot be metabolized in the human body. Apart from oxalic acid, the nephrotoxic glyoxylic acid concentration is high in all types of primary hyperoxaluria [15]. In type 1 primary hyperoxaluria, glycolic acid is also high, in type 2 glyceric acid concentration is high.

High plasma oxalic acid concentrations may also be caused by several disorders associated with fat malabsorption (non-inherited, secondary or enteric hyperoxaluria) and that may lead to kidney injury and insufficiency as well [10, 16, 17].

Both primary and enteric hyperoxaluria may be associated with kidney stone formation and with oxalate crystal deposition, CKD and kidney failure [16, 18]. There is a high oxalate nephropathy (recurrence) rate after kidney transplantation in patients with primary and enteric hyperoxaluria [10, 15, 16, 19, 20]. Apart from crystal deposition, oxalic acid and its precursor glyoxylic acid have been shown to cause inflammation and tubulotoxicity [12, 13]. This means that kidney damage may be caused without or before tubular crystal depositions occur.

Finally, high plasma oxalic acid concentrations may be caused by kidney insufficiency and failure *per se* since the main excretion pathway is glomerular filtration and tubular secretion [21,22]. As urinary oxalic acid concentrations are unreliable in CKD stage 4 and 5, in analogy to primary hyperoxaluria, plasma oxalic acid concentrations are used instead [23]. Oxalic acid is easily removed by hemodialysis, but rebounds to pre dialysis concentrations within 48 h [24]. Clearance of oxalic acid is highest in the first hours of dialysis. Residual diuresis is superior to dialysis in removing various (non-urea) solutes and even clinically negligible residual kidney function has been shown to provide non-urea solute clearance [25–29]. With decreasing residual diuresis, accumulation of waste products increases even further. This means that almost all pre-transplant patients have high plasma oxalic acid concentrations.

We hypothesize that DGF is associated with high pre-transplantation concentrations of waste products, such as oxalic acid and its precursors. Under unfavorable conditions, these may lead to inflammation, tubular toxicity and in worse cases even depositions in the transplanted kidney.

Residual diuresis was included in our study in order to exclude the possibility that the effect of oxalic acid and precursors may represent the effect of a whole collection of waste products that have accumulated as a result of reduced residual diuresis. In that case residual diuresis may be a better representative for the whole collection of waste products.

## Patients and Methods

All patients referred for kidney transplant work-up between September 2018 and January 2022 were asked to participate in this study. Follow-up was until January 1st^,^ 2023. The study conforms with the principles outlined in the Declaration of Helsinki. It was approved by the medical ethics committee of Erasmus University Medical Center Rotterdam, and all patients gave their written informed consent before inclusion (MEC 2018-044). Participation comprised a 10 mL blood sample drawn on the operation ward immediately before transplantation. Oxalic acid and substrates in the metabolic pathway of oxalic acid (precursors, see Figure 1); glyoxylic acid, glycolic acid and glyceric acid concentrations were determined. Residual diuresis (remaining urine volume) was based on the patient’s last reported 24 h urine volume submitted. Besides, a questionnaire on dietary habits was filled in. Results of this food frequency questionnaire will be described separately.

Kidney function related variables were collected. Recipient variables studied were: age and gender, body mass index (BMI), pre-transplant CRP, pre-transplant vPRA (% panel reactive antibody), use of diuretics (yes versus no), pre-transplant oxalic acid, glyoxylic acid, glycolic acid, glyceric acid concentrations, cardiac disease, diabetes mellitus, vascular disease, cerebrovascular event, previous kidney transplantations (yes versus no), kidney function replacement therapy (none/hemodialysis/peritoneal dialysis), and time between start dialysis and current transplantation (months). Donor and transplantation related variables were: donor type (living versus donation after brain death (DBD) and donation after cardiac death (DCD)), donor age, gender, serum creatinine, BMI, hypertension, diabetes mellitus, HLA mismatches (A, B, DR; 1–6), and cold ischemia period. Delayed graft function (DGF) was defined as dialysis treatment in the first week after transplantation.

To quantify the plasma organic acids (oxalic acid, glyoxylic acid, glycolic acid and glyceric acid), blood was drawn and placed on ice followed by centrifugation at 4°C without delay. Heparinized plasma samples were de-proteinized by addition of 75 µL 37% hydrochloric acid to 0.5 mL plasma followed by centrifugation. The supernatant was stored at −70°C until analysis. For quantification, a gas chromatography mass spectrometry (GC-MS) method was used.

### Data Analysis

Statistical analysis was performed using IBM SPSS Statistics 24. Baseline characteristics and outcomes were described as counts and percentages for categorical variables. For continuous variables, medians and interquartile ranges (IQR) were given for skewed continuous variables. Differences between continuous variables were studied using Mann-Whitney-U test. Differences between categorical variables were studied using Chi-square test.

Multivariable binary logistic regression analysis with backward elimination was used to study the influence of variables on the incidence of DGF, both in the total population (N = 496) and in the selection of patients on dialysis (n = 342). Kaplan Meier survival curves of the DGF and non-DGF populations were performed.

Spearman correlation analyses were performed to obtain correlation and 95%-confidence intervals between residual diuresis and plasma oxalic acid concentrations and residual diuresis and oxalic acid precursors. Correlation analyses were also performed for oxalic acid and its precursors and between precursors. Correlation values lower than 0.5 were considered weak. A *p*-value <0.05 was considered statistically significant.

## Results

512 patients consented and underwent a kidney transplantation. In 16 patients concentrations of oxalic acid and/or its precursors were missing. Results of 496 patients were available for analysis. Table 1 shows patient characteristics, there were no missing values in these 496 patients. Residual diuresis was 150 mL/day or less in 121 patients (24%). Table 2 shows donor and transplantation characteristics. There were 230 (46%) living donor transplantations, 88 (18%) donation after brain death transplantations and 178 (36%) donatio after cardiac death transplantations.

**TABLE 1:** Patient characteristics.

	Total population N = 496	no DGF n = 339	DGF n = 157	*p*-value no DGF versus DGF
Recipient characteristics				
Gender male n (%)	300 (60.5)	197 (58.1)	103 (65.6)	0.068
Age (years) median (IQR)	62 (51; 69)	60 (49; 68)	64 (55; 70)	0.008
BMI median (IQR)	27 (24; 31)	26 (23; 30)	30 (25; 34)	<0.001
CRP mg/L median (IQR)	3 (1; 8)	3 (1; 6)	5 (2; 12)	<0.001
Medical history				
Cardiac event n (%)	89 (17.9)	51 (15.0)	38 (24.2)	0.010
Cerebrovascular accident n (%)	60 (12.1)	38 (11.2)	22 (14.0)	0.227
Vascular event n (%)	43 (8.7)	22 (6.5)	21 (13.4)	0.011
Diabetes mellitus n (%)	167 (33.7)	88 (26.0)	79 (50.3)	<0.001
Residual diuresis in mL/day median (IQR)	1000 (200; 2000)	1500 (500; 2000)	250 (0; 875)	<0.001
Use of diuretics, yes n (%)	184 (37.1)	128 (37.8)	56 (35.7)	0.365
Dialysis n (%)				<0.001
No	154 (31.0)	152 (44.8)	2 (1.3)	
PD	105 (21.2)	78 (23.0)	27 (17.2)	
HD	237 (47.8)	109 (32.2)	128 (81.5)	
Time between last dialysis and transplantation (days)				
PD only median (IQR)	0.39 (0.21; 0.71)	0.39 (0.23; 0.90)	0.35 (0.20; 0.59)	0.217
HD only median (IQR)	1.34 (0.80; 1.95)	1.33 (0.98; 2.09)	1.32 (0.64; 1.77)	0.035
Time on dialysis in months median (IQR)	15(0; 30)	6.8 (0; 21.8)	28 (16; 44)	<0.001
Hyperoxaluria, non-renal cause n (%)	20 (4.0)	7 (2.1)	13 (8.3)	0.002
Oxalic acid in μmol/L median (IQR)	33 (18; 57)	25 (14; 48)	46 (32; 64)	<0.001
Glycolic acid in μmol/L median (IQR)	5.7 (5.0; 6.7)	5.5 (4.8; 6.4)	6.0 (5.3; 7.0)	<0.001
Glyoxylic acid in μmol/L median (IQR)	2.0 (1.4; 2.8)	1.8 (1.2; 2.5)	2.3 (1.7; 3.3)	<0.001
Glyceric acid in μmol/L median (IQR)	2.6 (2.2; 3.1)	2.4 (2.1; 2.8)	2.9 (2.5; 3.4)	<0.001
vPRA median (IQR)	4 (0; 5)	4 (0; 5)	4 (0; 22)	0.055
vPRA n (%)				0.163
<4	229 (46.2)	166 (49.0)	63 (40.1)	
4–84	230 (46.4)	150 (44.2)	80 (51.0)	
≥85	37 (7.5)	23 (6.8)	14 (8.9)	
First kidney transplantation n (%)	421 (84.9)	297 (87.6)	124 (79.0)	0.010

IQR, interquartile range; HD, hemodialysis; CAPD, continuous ambulatory peritoneal dialysis; vPRA, virtual panel reactive antibodies.

**TABLE 2:** Donor and transplantation characteristics.

	Total population N = 496	no DGF n = 339	DGF n = 157	*p*-value no DGF versus DGF
Donor characteristics				
Donortype				<0.001
Living donor n (%)	230 (46.4)	220 (64.9)	10 (6.4)	
DBD n (%)	88 (17.7)	57 (16.8)	31 (19.7)	
DCD n (%)	178 (35.9)	62 (18.3)	116 (73.9)	
Age donor years median (IQR)	58 (48; 67)	56 (48; 65)	62 (51; 69)	0.003
Donor gender male n (%)	253 (51.0)	159 (46.9)	94 (59.9)	0.005
Donor BMI median (IQR)	26 (23; 29)	26 (23; 29)	26 (24; 29)	0.445
Donor comorbidity				
hypertension n (%)	155 (31.3)	99 (29.2)	56 (35.7)	0.091
Diabetes mellitus n (%)	23 (4.6)	12 (3.5)	11 (7.0)	0.073
Donor creatinine (μmol/L) median (IQR)	72 (59; 83)	73 (63; 83)	66 (53; 84)	0.006
Transplantation characteristics				
HLA A, B, DR mismatches				0.362
0–3	296 (59)	200 (59)	96 (61.1)	
4–6	6200 (40.3)	139 (41.0)	61 (38.9)	
HLA mismatches median (IQR)	3 (3; 6)	3 (2; 5)	3 (2; 4)	0.660
Cold ischemia time (min)	389 (120; 707)	136 (111; 493)	693(534; 797)	<0.001

IQR, interquartile range; DBD, donation after brain death; DCD, donation after cardiac death; HLA, Human leukocyte antigen.

Table 3 shows the concentrations of oxalic acid, glycolic acid, glyoxylic acid, and glyceric acid in the total patient population, the pre-dialysis population and in the population on dialysis. Only 1.2% of the patients had pre-transplant oxalic acid concentrations within the normal range. All glyoxylic acid concentrations were above the upper limit of normal. Glycolic acid and glyceric acid concentrations were within the reference range in 87% and 22% of cases respectively. Patients on dialysis had significantly higher oxalic acid, glycolic acid, glyoxylic acid and glyceric acid concentrations compared to pre-dialysis patients.

**TABLE 3:** Median values of oxalic acid, glycolic acid, glyoxylic acid and glyceric acid in the total study population and subgroup population on dialysis and not on dialysis.

	Normal values	Total study population (N = 496)	Subgroup population not on dialysis (n = 154)	Subgroup population on dialysis (n = 342)	*p*-Value
Oxalic acid in µmol/L *median (IQR)*	2.5–7.0	33.1 (18.0; 56.6)	14.5 (11.3; 21.0)	45.8 (30.1; 65.0)	<0.001
Glycolic acid in µmol/L *median (IQR)*	3.6–7.6	5.7 (5.0; 6.7)	5.0 (4.5; 5.6)	6.0 (5.3; 7.0)	<0.001
Glyoxylic acid in µmol/L *median (IQR)*	0.2–0.4	2.0 (1.4; 2.8)	1.3 (1.0; 1.8)	2.3 (1.7; 3.3)	<0.001
Glyceric acid in µmol/L *median (IQR)*	1.3–2.1	2.6 (2.2; 3.1)	2.2 (2.0; 2.5)	2.8 (2.4; 3.3)	<0.001

IQR: interquartile range.

*p* values measured with Mann-Whitney U test for the difference between the subgroups of patients on dialysis and patients not on dialysis.

**FIGURE 2:**
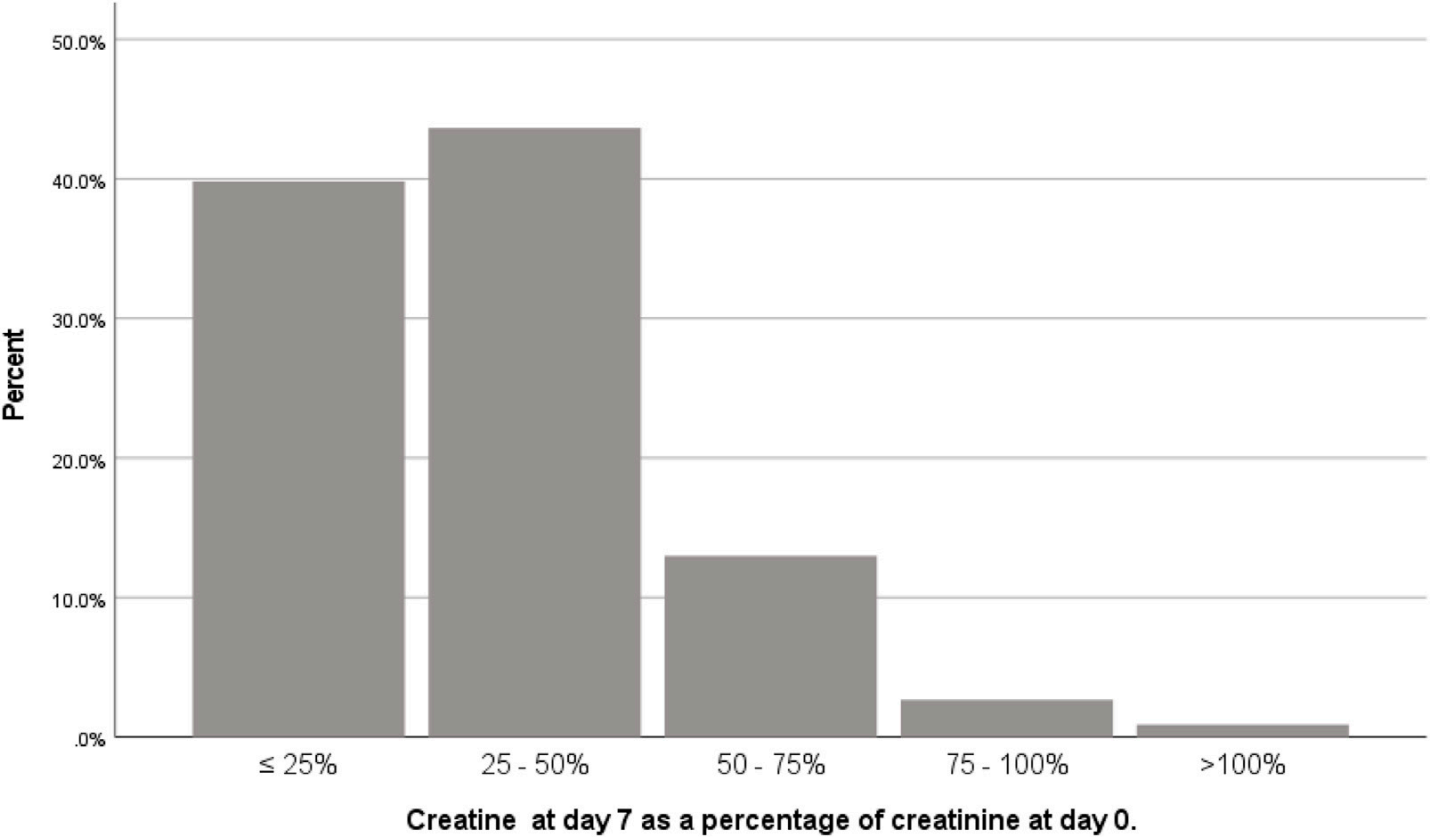
Ratio of serum creatinine on day 7 after transplantation and serum creatinine on day of transplantation in patients without delayed graft function (n = 339).

Delayed graft function occurred in one-third (n = 157; 32%) of the population. There were 339 patients without DGF. In 84% of patients without DGF, serum creatinine at day 7 was at least halved compared to pre-transplant serum creatinine (Figure 2). Only 3 patients had an increase of serum creatinine on day 7 compared to day 0. One of them had a surgical complication with temporary increase in serum creatinine on day 7. Consequently, two patients without the diagnosis DGF that had an increase in serum creatinine on day 7, but adequate residual diuresis, ruling out the necessity for dialysis. Tables 1, 2 show that there are large differences between the populations with versus without DGF. The influence on the DGF risk of all variables shown in Tables 1, 2 was tested in binary logistic regression analysis. In univariable analysis, recipient variables with significant effect on DGF risk were: age, BMI, CRP, cardiac event, vascular disease, diabetes mellitus, residual diuresis, dialysis type, dialysis vintage, oxalic acid, glyoxylic acid and glyceric acid concentration, and number of previous kidney transplants. Donor variables with significant influence in univariable analysis were: donor type, age, gender, and cold ischemia time. In multivariable analysis, after backward elimination, categorical variables that remained in the model with a significant influence on the DGF risk were: donor type, and dialysis type. Besides, recipient BMI, donor age, donor serum creatinine, and glyoxylic acid concentration were significantly and directly proportionally related to the DGF risk, while residual diuresis and glycolic acid concentration were inversely proportionally related the DGF risk (Table 4). There was no interaction between any combination of residual diuresis, glyoxylic acid, and glycolic acid. There was no interaction between donor type and glyoxylic acid concentration, glycolic acid concentration, dialysis type, residual diuresis, and recipient BMI.

**TABLE 4:** Multivariable binary logistic regression analysis on delayed graft function, using backward elimination. Total population N = 496, events = 157.

	Exp(B)	95% C.I.for EXP(B)		Sig
		Lower	Upper	
Donor type (living)				<0.001
DBD	6.695	2.635	17.015	<0.001
DCD	33.580	14.379	78.422	<0.001
Dialysis type (none)				<0.001
Hemodialysis	37.621	7.229	195.786	<0.001
Peritoneal dialysis	11.532	2.192	60.659	0.004
Recipient BMI (kg/m^2^)	1.099	1.041	1.160	0.001
Residual diuresis (per 100 mL)	0.939	0.900	0.979	0.003
Donor age (years)	1.030	1.009	1.053	0.006
Donor creatinin (µmol/L)	1.010	1.002	1.018	0.010
Glycolic acid (µmol/L)	0.884	0.800	0.976	0.015
Glyoxylic acid (µmol/L)	1.120	1.022	1.227	0.015
HLA mismatches	0.819	0.664	1.009	0.061
Donor gender (male)	0.583	0.321	1.056	0.075
Constant	0.000			0.000

The same analysis was performed in the population restricted to patients on dialysis (n = 342). Glyoxylic acid concentration failed significance, but all other variables with a significant influence in the total population also had a significant influence in the population on dialysis (Table 5).

**TABLE 5:** Multivariable binary logistic regression analysis on delayed graft function, using backward elimination. Dialysis population n = 342, events = 155.

	Exp(B)	95% C.I.for EXP(B)		Sig
		Lower	Upper	
Donor type (living)				<0.001
DBD	6.031	2.385	15.248	<0.001
DCD	36.257	15.215	86.398	<0.001
Dialysis type (HD)	0.336	0.172	0.657	0.001
Recipient BMI (kg/m^2^)	1.119	1.057	1.184	<0.001
Residual diuresis (per 100 mL)	0.993	0.989	0.997	0.001
Donor creatinin (µmol/L)	1.012	1.004	1.020	0.004
Donor age (years)	1.027	1.006	1.049	0.013
Glycolic acid (µmol/L)	0.889	0.804	0.983	0.021
Glyoxylic acid (µmol/L)	1.087	0.993	1.191	0.071
Constant	0.001			0.000

DBD, donation after brain death; DCD, donation after cardiac death; HD, hemodialysis.

**FIGURE 3:**
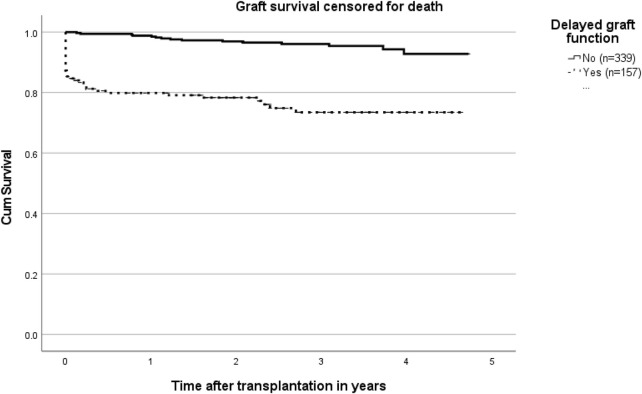
Kaplan Meier curves on graft survival censored for death in patients with and without delayed graft function (*p* < 0.001).

In a follow up period of almost 5 years, the survival curve shows significantly worse results in patients with DGF compared to those without DGF (*p* < 0.001; Figure 3).

The relationship between residual diuresis and oxalic acid and its precursors was studied using Spearman’s correlation. It showed a significant, moderate correlation between oxalic acid and residual diuresis (N = 496; r = −0.529; *p* < 0.001). Correlation of residual diuresis with glycolic acid (r = −0.287; *p* < 0.001); with glyoxylic acid (r = −0.258; *p* < 0.001); and with glyceric acid (r = −0.260; *p* < 0.001) was weak but significant.

The relationship between oxalic acid and its precursors was studied using Spearman’s correlation. Correlation of oxalic acid with glyoxylic acid (r = 0.685; *p* < 0.001); and with glyceric acid (r = 0.570; *p* < 0.001) was significant. Correlation of oxalic acid with glycolic acid (r = 0.472; *p* < 0.001) was weak, but statistically significant.

**FIGURE 4:**
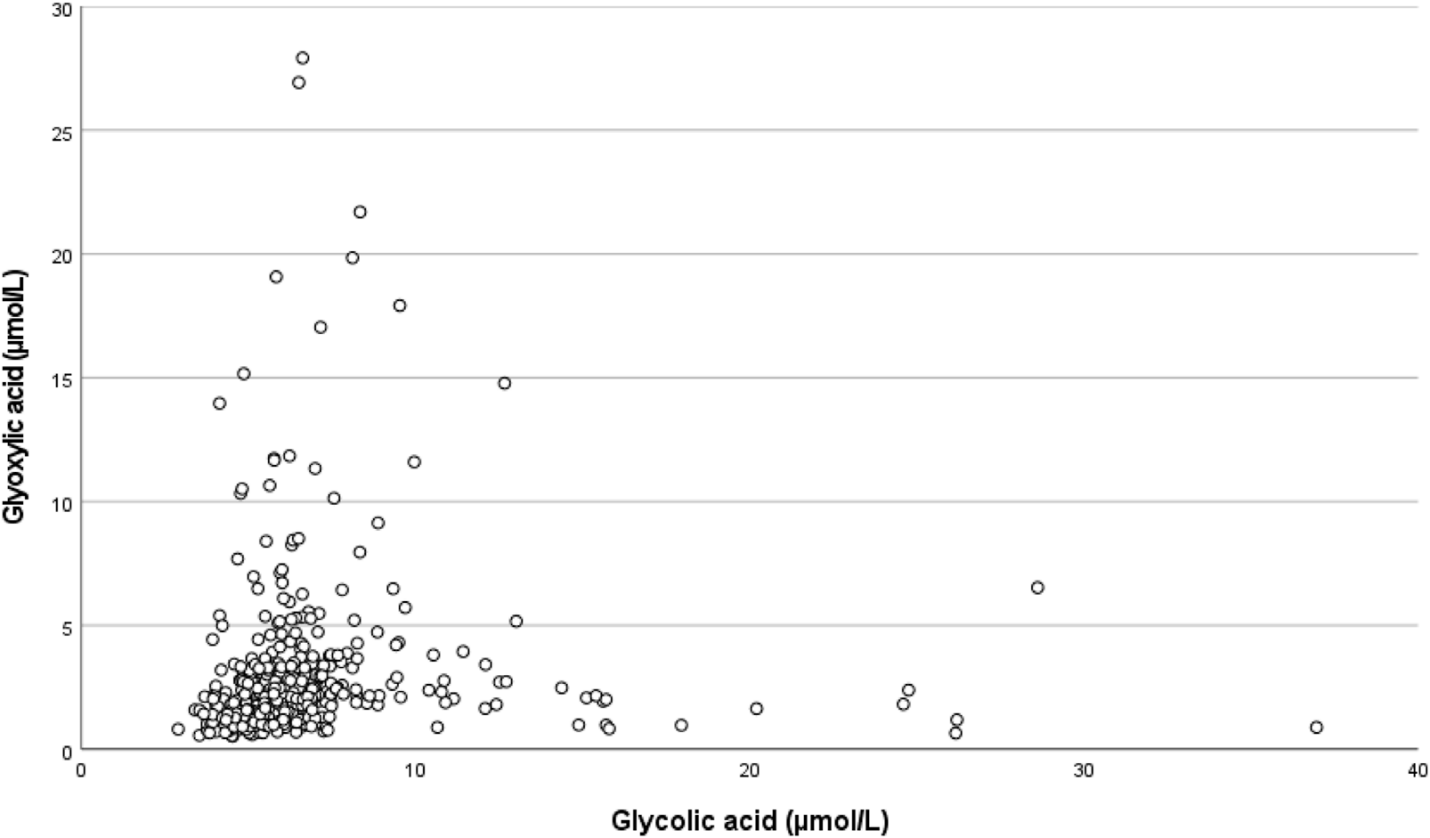
Scatterplot showing the relationship between pre-transplant glyoxylic acid concentration and glycolic acid concentration.

Because glyoxylic acid significantly increased the DGF risk and glycolic acid decreased that risk in our multivariable regression analysis, their relationship was studied. Correlation of glyoxylic acid and glycolic acid was weak, but statistically significant (r = 0.370; *p* < 0.001). The scatterplot showed a dichotomy: In the extremes of the graph patients had either high glyoxylic acid or high glycolic acid concentrations, not both (Figure 4).

## Discussion

Our study shows a significant effect of residual diuresis on the incidence of DGF after kidney transplantation: this holds true for the total population, but also, after exclusion of pre-dialysis patients, for the population on dialysis. Most probably, the association between low residual diuresis and DGF is the result of the accumulation of more or less toxic waste products that were not adequately removed via dialysis when residual diuresis decreased. Our study confirms that DGF is associated with decreased long-term graft survival [1, 5, 30].

There are two studies on the incidence of DGF after kidney transplantation, that also describe a significant influence of residual diuresis [30, 31]. Chaumont et al. studied the incidence of DGF in their center and concluded that perioperative saline loading and higher residual diuresis attributed to a lower risk [30]. Jahn aimed at risk factors for DGF and 1 year graft failure and concluded that residual diuresis influenced the DGF risk [31]. In patients on peritoneal dialysis [27, 32] and hemodialysis [33], patient survival has been shown to be negatively influenced by low residual diuresis. Besides, in dialysis patients, decreasing residual kidney function is associated with serious comorbidities [33–41]. This means that the pre-transplant patients with low or absent residual diuresis, are the less vital patients compared to those with significant residual diuresis volume. The cause of comorbidities is probably associated with accumulation, toxicity and/or deposition of toxic waste products, left behind as a result of failing diuresis [25, 28, 29, 42, 43]. Sudden excretion of these products by the newly transplanted kidney might cause inflammation, toxicity and possibly even depositions, that lead to kidney injury, and to impaired kidney function or even inhibition of the onset of donor kidney function.

Our study also shows that oxalic acid and its direct precursors glyoxylic acid, glycolic acid and glyceric acid are examples of waste products that accumulate when the kidney fails. In many pre-transplant patients, plasma oxalic acid concentrations are comparable to those of patients with primary hyperoxaluria. The glycolic acid concentrations are above the upper normal value in only 13% of cases (Table 3). Recently a small scale study showed normal glycolic acid concentrations in the dialysis population [44].

There was a significant and relevant correlation between oxalic acid concentration and glyoxylic and glyceric acid concentrations implicating that when high oxalic acid concentrations are found in pre-transplant patients, relatively high glyoxylic and glyceric acid concentrations may be expected. Highest concentrations of oxalic acid, glyoxylic acid, glycolic acid and glyceric acid were found in dialysis patients compared to pre-dialysis patients.

Both oxalic acid and its direct precursor glyoxylic acid are known for their tubulotoxicity, [12, 13, 45]. Although concentrations of both oxalic acid and glyoxylic acid significantly influenced DGF risk in univariable binary logistic regression analysis, in multivariable analysis only glyoxylic acid remained in the model and significantly influenced the DGF risk. This effect was independent of the effect of residual diuresis, emphasizing the individual toxic effect of glyoxylic acid. Glycolic acid on the other hand exerted a protective effect. Glycolic acid is not toxic. There is an inverse relationship between glyoxylic acid and glycolic acid as shown in Figure 4. The “protective” effect may be the result of shifting to non-toxic glycolic acid instead of toxic glyoxylic acid (Figure 1). On the other hand, in the restricted dialysis population glyoxylic acid failed significance, possibly as a result of lower numbers of patients included. When residual diuresis was removed from the model, the influence of glyoxylic acid became significant (*p* = 0.017), indeed suggesting that residual diuresis is a surrogate marker for at least glyoxylic acid, but probably also for many other toxic waste products.

A limitation of our study is that residual kidney function was not available in patients with residual diuresis, thus residual urine volume was used as a representative instead. Residual urine volume was based on the patient’s last 24-h urine collection submitted. Last collection may have been a few months before transplantation as 24-h urine collection must be submitted every 3 months for clinical care. Dialysis patients are aware of their 24 h urine production as it determines their fluid restriction.

Another limitation is the definition used for DGF, which is the most commonly used: dialysis in the first week after transplantation. However, fluid overload as the indication for dialysis may not be set in patients with preserved residual diuresis. When assessing transplant function on day 7 post-transplantation, there were only 2 patients without the diagnosis DGF that had an increase in serum creatinine, but adequate residual diuresis, ruling out the necessity for dialysis. All other patients without DGF had a decrease in serum creatinine. Without dialysis, even a small creatinine decrease of 10% is supposed to be the result of function of the transplanted kidney. This means that the definition for DGF turns out to be adequate in our population.

Preservation of residual diuresis, even after start dialysis, is useful and gains attention nowadays. Dietary and pharmacological interventions are defined to ensure optimal native kidney function preserving care [46]. Besides, on top of Kt/V there should be more attention for removal of other waste products, because high concentrations have a negative effect on graft function. This means that more intensive or optimal dialysis treatment, could have a beneficial effect on the prevention of DGF after transplantation. Besides, our study adds an argument to stimulate pre-emptive transplantation in patients who still have adequate diuresis and relatively low concentrations of waste products and thus are in relatively good condition.

## Data Availability

The raw data supporting the conclusions of this article will be made available by the authors, without undue reservation.

## Abbreviations

AGTX, Alanine glyoxylate aminotransferase; CAPD, Continuous ambulant peritoneal dialysis; DGF, Delayed graft function; DBD, Donation after brain death; DCD, Donation after cardiac death; eGFR, estimated Glomerular filtration rate; GO, Glycolate oxidase; HD, Hemodialysis; HLA, Human leucocyte antibody; IQR, interquartile range; LDH, Lactic dehydrogenase; vPRA, virtual Panel reactive antibodies.
